# Past, Present, and Future: A Review of Uterus Transplant

**DOI:** 10.3389/ti.2025.15325

**Published:** 2025-12-01

**Authors:** Liza Johannesson, Connor Fischbach, Olivia Walker, Giuliano Testa

**Affiliations:** Division of Abdominal Transplantation, Annette C. and Harold C. Simmons Transplant Institute, Baylor University Medical Center, Dallas, TX, United States

**Keywords:** transplant, absolute uterine factor infertility, mayer-rokitansky-kuster-hauser syndrome (MRKH), female infertility, uterus transplant

## Abstract

Since the first live birth in 2014 after uterus transplantation, the procedure has become a viable fertility treatment worldwide for the 1 in 500 women affected by absolute uterine factor infertility. In this review, we provide insight on Mayer-Rokitansky-Kuster-Hauser syndrome (MRKH) and the other conditions that lead to the development of AUFI. Additionally, we provide a comprehensive overview of the evolution of uterus transplantation from the first sporadic cases to the current clinical status of the procedure, and detail multiple aspects that go into a successful UTx. Furthermore, we review some of the more recent developments in this rapidly expanding field and evaluate the prospective direction of UTx.

## Introduction

**FIGURE 1:**
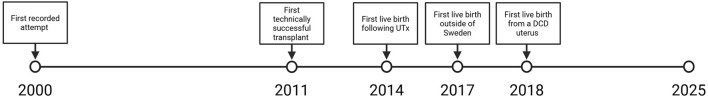
Significant moments in uterus transplantation.

With the advancement of knowledge and technology in the fields of transplant, gynecology and reproductive endocrinology, Uterus transplant (UTx) has emerged as a new type of fertility treatment that provides the 1 in 500 women affected by absolute uterine factor infertility (AUFI) a viable path towards parenthood. Uterus Transplant is unique insofar as it is the only solution to AUFI that allows the experience of pregnancy and delivery. Since the first live birth following UTx in 2014, the field of UTx has rapidly developed as shown in Figure 1 and has become an option for family planning in multiple countries [1]. While UTx has grown significantly, the field is still in its infancy, making it imperative to evaluate the many aspects that go into a successful transplant and, ultimately, the birth and development of a child born from a mother recipient of a uterus transplant. In this review, we discuss the path that led to the development of UTx, the most recent developments in the field, and its future directions.

## Absolute Uterine Factor Infertility

Infertility due to uterine factor is either congenital or acquired. The acquired form can be caused by a previous hysterectomy or by conditions making the uterus incapable of embryo implantation or completion of pregnancy. Conditions affecting the uterus reproductive ability can be cavital, such as, Asherman syndrome which presents as significant scarring of the endometrial lining caused by severe postpartum hemorrhage or endometrial infection, or myometrial, such as, fibroids that can lead to distortion of the uterine cavity and affect implantation [2]. Uterine fibroids can be identified in 20–40 percent of reproductive aged women and present in up to 27 percent of patients seeking reproductive assistance [3, 4]. Additional conditions affecting uterus functionality include uterine septa which are present in roughly 2 to 3 percent of the general population and are associated with poor pregnancy outcomes [5]. Hysterectomy is one of the most common surgical procedures performed in women in the United States, totaling around 600,000 per year [6]. Most hysterectomies are performed for benign conditions such as myomas, abnormal uterine bleeding and endometriosis, with only ∼10% performed as treatment for cancer [6].

## Mayer-Rokitansky-Kuster-Hauser Syndrome (MRKH)

Congenital uterine agenesis (MRKH) is to date the most common indications for UTx. The exact underlying genetic aspects are not yet fully understood. However, some have suggested that this condition is autosomal dominant with incomplete penetrance, although this hypothesis has been challenged [7–11].

Women with this diagnosis have a genetic karyotyping of 21 females with uterovaginal agenesis and typical secondary sexual characteristics and XX Chromosomes [12]. MRKH can be sorted into two different types [13–15]. Type II MRKH often presents with renal abnormalities such as renal agenesis or a pelvic kidney [16]. However, both types present with significant agenesis/aplasia of the uterus and upper portion of the vagina leading to AUFI. AUFI has significant implications for the psychological wellbeing of those affected. Women with MRKH scored significantly higher on questionnaires for anxiety, depression, eating disorders, and low self-esteem [17]. Additional studies have indicated significant impairment of mental-health-related quality of life and generally poorer mental health in MRKH patients when compared to controls [18, 19]. Furthermore, the interviewing process for UTx has revealed that AUFI and MRKH have significant impact on self-perception and the relationships of those affected [20].

## Early Uterus Transplantation

The first published human UTx attempt occurred in Saudi Arabia in 2000 [21]. The living donor graft had to be removed 3 months post-transplant due to thrombosis and necrosis. This initial attempt generated interest worldwide and represented a major event in the field of UTx despite not resulting in a live birth. The next reported UTx was performed in Turkey in 2011 from a deceased donor [22]. For many years this case was considered a technical success with a viable graft but lacking a successful reproductive outcome. Nine years after the transplant, in 2020, the recipient had a live birth [23]. Both these two initial cases are representative of the challenges and coordination required in the time frame between transplant and the live birth of a child.

In 2014, the first live birth of a child following UTx was reported from Sweden [1]. This case was proof of concept for the procedure and would ultimately establish the definition of a successful UTx [1]. The patient was a 35-year-old woman with type 2 MRKH who received a UTx via directed living donation [1]. She had a single embryo transfer 1-year post-transplant that resulted in pregnancy [1]. A male baby was delivered prematurely at 31 weeks and 5 days via cesarean section. This first live birth was preceded and made possible by over a decade of extensive research in animal models and well-established protocols [24–27]. In the results of the rest of the Swedish clinical trial six women gave birth to nine children. The live birth weight per successful transplant was 67% [28]. Additionally, none of the children born were undersized for gestational age [28].

### Expansion of UTx

The first live birth after UTx served as a catalyst for further growth in the field of UTx. Several transplant centers around the world began to establish clinical UTx trials. In 2016, two programs started in the United States (Cleveland Clinic in Cleveland, Ohio; Baylor University Medical Center, Dallas) [29]. The Dallas group [Dallas Uterus Transplant Study (DUETS)] became the first group in the world to replicate the success of a live birth after UTx of the Swedish transplant team in 2017 [30]. The first 20 cases were performed as an IRB study (2016–2019) and resulted in 17 live births [31]. The remarkable outcome of this study was that when the transplant was a technical success (viable graft 30 days post surgery), 100% of cases had at least one live birth. This study aided in proving the reproductive potential of the transplanted uterus [31]. The results of this study have helped considerably in adding to the existing knowledge in the field and in developing protocols in UTx. Currently, there are four active UTx programs in the United States (Cleveland Clinic in Cleveland, University of Pennsylvania, UAB, Baylor University Medical Center) and the added volume in cases and live births correspond to approximately 60% of the volume worldwide [29].

While DUETS was underway, researchers in other countries began assessing the feasibility of UTx in their transplant centers as well. In South America, Brazil is currently the only country with reported attempts and a reported live birth. The live birth in Brazil also represented another significant clinical first for UTx as it was the first live birth following the transplantation of a deceased donor’s uterus [32]. In this case, immunosuppression was induced with prednisone and thymoglobulin [32]. Immunosuppression was maintained via tacrolimus and mycophenalate mofetil (MMF) until 5 months post-transplant in which azathioprine replaced MMF [32]. The recipient’s first menstruation occurred 37 days post-transplant, and embryo transfer occurred 7 months post-transplant [32]. Following the embryo transfer, a baby girl was delivered at 36 weeks gestation. At birth, the baby weighed 2,550 g [32]. Remarkably, no episodes of rejection occurred post-transplant and graft hysterectomy was performed at delivery [32].

In Europe, the second country to begin a clinical trial for UTx was the Czech Republic [33]. In their initial experience, 7 of 10 attempts resulted in a successful transplant. The results saw three pregnancies which would ultimately lead to the live birth of two children [34]. Additionally, the initial Czech experience provided further evidence for the viability of deceased donors in UTx with one of the children being born in a recipient with a graft procured from a deceased donor [33]. Following the Czech clinical trial, clinical trials and initial attempts at UTx would take place in France, Germany, Italy, and the United Kingdom [35–38]. Currently, there no documented case reports or case series detailing the results seen in UTx recipients in France. In the German trial, 4 women received a uterus transplant from directed living donors with the fifth attempt being stopped due to the discovery of insufficient vasculature of the prospective graft during back table preparation Two of the women would go on to give birth at 35 weeks and 36 weeks with both of the children born being in the 15th percentile for birthweight [36]. In the Italian clinical trial, investigators performed two transplants using deceased donors with one of the transplants resulting in graft loss due to thrombosis and the other resulting in a live birth [37]. The live birth was delivered via cesarean section at 34 weeks and weighed 1725 g at birth [37]. In the United Kingdom, there is a published case report detailing a successful transplant attempt [38]. However, there is no indication of whether or not the transplant resulted in the live birth of a child.

**FIGURE 2:**
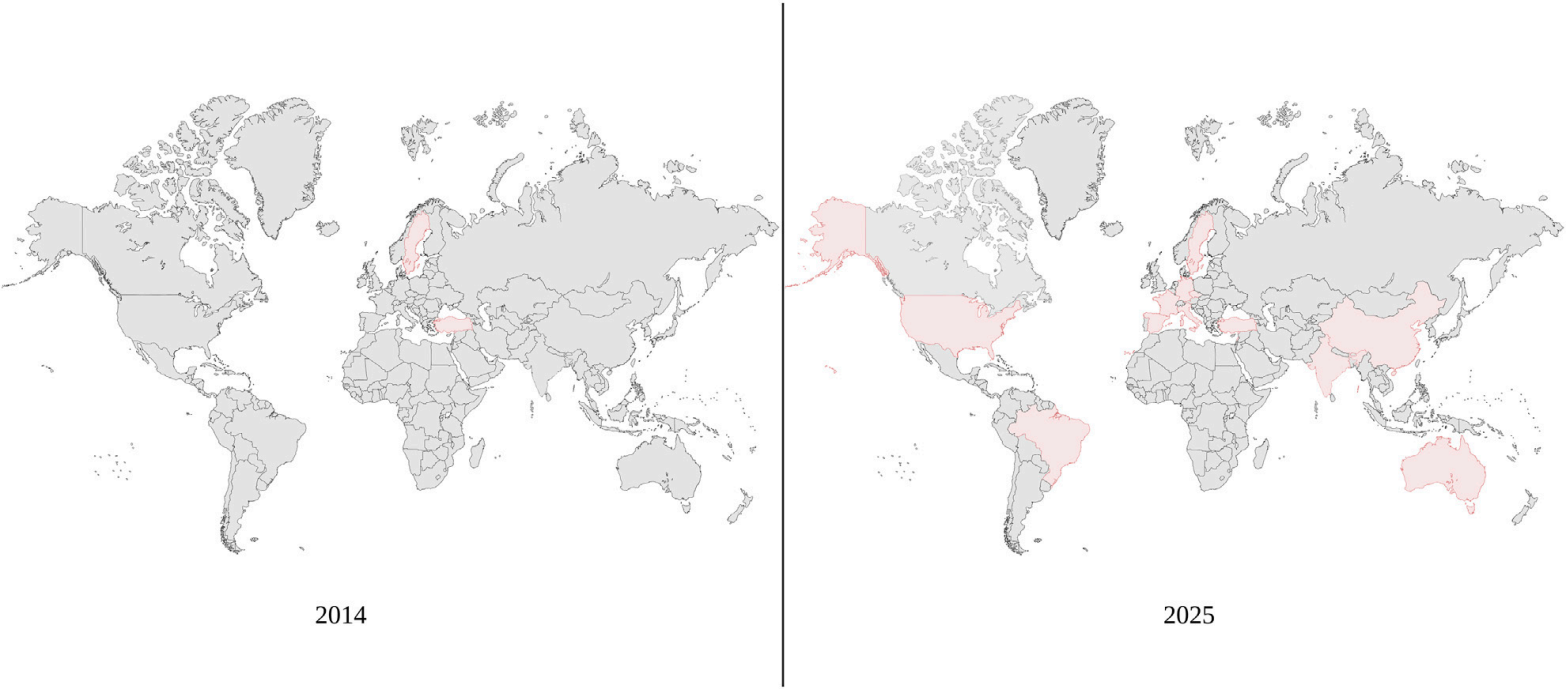
Global expansion of uterus transplantation. Since 2014, uterus transplantation has expanded to more countries and become more prominent globally.

In Asia, there have been reported attempts of UTx using living donors in both China and India [39, 40]. Notably, the case report in China documents the first use of robotic assistance in the procurement of a uterine graft [39]. In this attempt, the living donor was a 42-year-old woman who had two previous vaginal deliveries. Following the successful transplant, the recipient had their first menstrual cycle 40 days post-transplant [39]. The experience in India provided additional support of the viability of UTx. In the Indian attempts, both transplants were successful and the recipients had their first menstrual cycle at 34- and 48-day post-transplant [40]. In Australia, there are two established UTx programs with the first Australian live birth occurring in 2024 [41]. More recently, UTx has expanded into Singapore. In this instance, a living donor was used for the operation and the transplant was a technical success with the recipient having their first menstruation 38 days post-transplant [42]. As UTx has expanded on a more global scale, the International Society of Uterus Transplantation (ISUTx) was founded in 2016 [43]. Figure 2 displays how UTx has expanded globally since the first live birth in 2014.

## Present Day Uterus Transplantation

**FIGURE 3:**
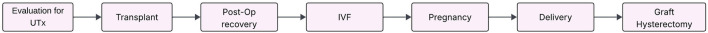
Stages of uterus transplant. Prospective patients must undergo evaluation for UTx per the transplant center’s protocol to determine if candidate. Once a patient is cleared, transplant surgery will be scheduled in coordination with living donor or tentatively planned pending deceased donor. The recipient’s surgery takes 5 or 6 h with an average hospital stay of 6 days to follow. Depending on patient’s post-op recovery period, initial IVF embryo transfer occurs between 2–7 months post-op. Additional rounds of IVF may be necessary to achieve pregnancy. UTx recipient pregnancies have proven to result in third-trimester live births, at which point the baby will be delivered via cesarean section. Graft hysterectomy may occur at the time of delivery or at a later time depending on the UTx recipient preference. UTx recipients have been able to safely carry two pregnancies, so if cleared, the recipient may go through additional rounds of IVF.

As UTx has become more prevalent, there has been a push to standardize the various aspects of the procedure shown in Figure 3, such as the evaluation process, operational procedures, post-op recovery, IVF, and delivery. Standardization of UTx allows for better outcomes for the donor and recipient, better graft viability, and ultimately more live births. The collaboration between researchers has been pivotal for the continued development of the field and remains paramount as the field of UTx continues to evolve. As the field of UTx has evolved, so have many of the important aspects of the procedure.

### Recipient Evaluation for Uterus Transplantation

The age range for potential recipients in centers performing UTx is typically set to childbearing age (18–40 years), with the upper limit being set to reduce the risk of potential pregnancy complications and to ensure oocyte quality [37]. For a majority of centers, the recipient inclusion and exclusion criteria were similar to the position statement on UTx released by the American Society for Reproductive Medicine, which in summary required no severe medical comorbidities and a body mass index less than 30 [44]. In addition to having no severe medical comorbidities, centers often completed extensive interviews and psychological evaluations before proceeding forward with the selection of a candidate for UTx. The intensive interviews and psychological screening allow for care providers to adequately assess current mental health, adequate social support, and adaptive coping skills necessary to deal with the numerous stressors that go along with the transplant surgery, the possible side effects of the immunosuppressive therapy, the uncertainty of successful embryo transfer and the potential complications of pregnancy and delivery [45–47]. Overwhelmingly, the results of these interviews and psychological assessments revealed unique insights into the motivation behind seeking UTx and how AUFI can mentally impact the women it afflicts. One of the most common motivations for seeking UTx amongst potential recipients was often the desire to experience gestation [48–50]. Other common reasons included wanting to defy the odds and the desire to have a biological child [48, 50].

### Donor Selection and Care Post Uterus Transplantation

In UTx, utilization of deceased and living donor grafts has been proven to be equally successful [32]. Nonetheless, both options have their respective challenges. A deceased donor graft eliminates the risk of surgical and psychological complications that can arise with a living donor and gives an opportunity to access extended graft vascularity [51]. However, several logistical aspects require a substantial amount of planning. Utilization of a deceased donor is restricted by donor availability due to UTx currently requiring a brain-dead donor. As a result, uterus grafts from deceased donors have been reported to have limited availability in multiple countries [52–54]. Another logistical issue present with uterus procurement from deceased donors is the lack of standardized evaluation criteria, which reduces the ability to extensively screen the donor for abnormal pap smears, absence of major abdominal or pelvic surgery, history of donor infertility/subfertility, human papillomavirus, and other relevant systemic disease limiting the knowledge regarding the quality of the graft [52]. Furthermore, the recipient and her family may have to relocate to an area close to the hospital for an extended period of time, which may result in increased psychological stress [52, 55].

The utilization of a living donor graft involves a major elective surgery on a healthy woman, without direct benefits to herself, and with potential risks. While the use of robotic hysterectomy has made substantial strides in reducing risk, the overall risk is not zero [56]. The most commonly seen complication is ureteric injury, this has been seen in both robotic assisted approach and the open laparoscopic approach [21, 33, 57]. Nonetheless, similar to other types of living donation, there remains the risk of infection and even death. Thorough assessment of living donors prior to surgery and transparency of the potential risks involved is paramount when a living donor donates.

### Surgical Aspects of Uterus Transplantation

**FIGURE 4:**
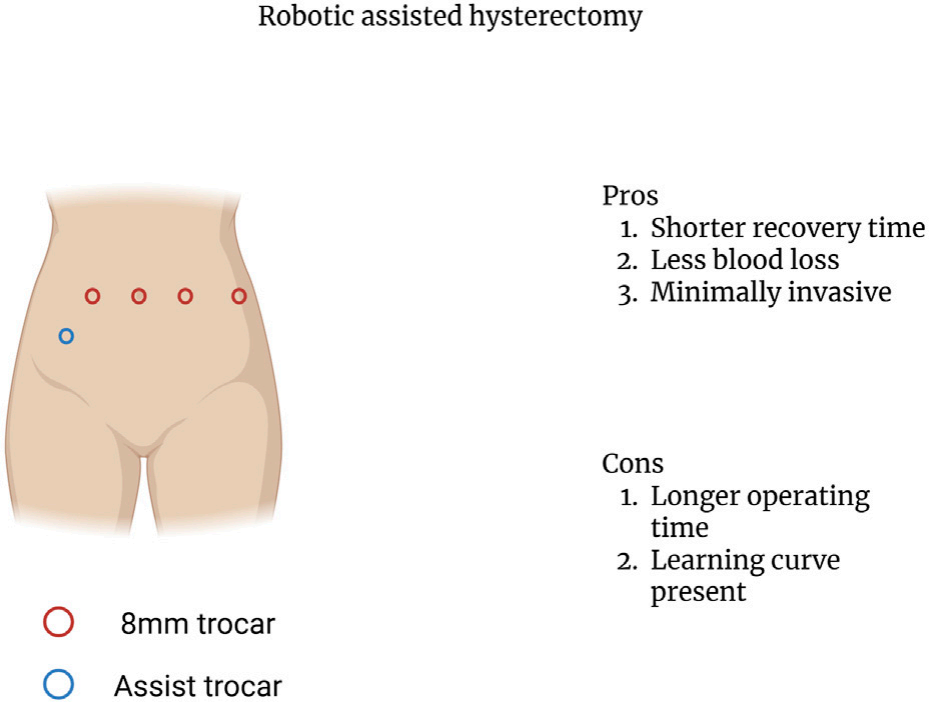
Robotic assisted hysterectomy trocar insertion points.

The surgical aspects of UTx can be broken down into three separate components: donor hysterectomy, graft implantation, and graft hysterectomy. The first part, the donor hysterectomy was initially performed through open laparotomy. However, the introduction of robotic assisted techniques in several centers has been shown to be beneficial [56]. During the donor hysterectomy, the vascular pedicles of the uterus must be recovered to ensure graft inflow and outflow [58]. The uterine artery in conjunction with the whole trunk of internal iliac artery or only the anterior branch is utilized to provide inflow with the inferior and superior uterine veins being used to establish outflow [58–60]. In living donors, the use of robotic assisted approaches has been shown to result in lower estimated blood loss, decreased hospital stay, and decreased length of sick leave when compared to the open approach [61]. In addition, the use of robotic assisted techniques has demonstrated better graft viability [56]. Furthermore, the use of robotic assisted techniques allows for better operative visibility and greater intraoperative maneuverability while minimally invasive with the points of trocar insertion being illustrated in Figure 4 [62].

**FIGURE 5:**
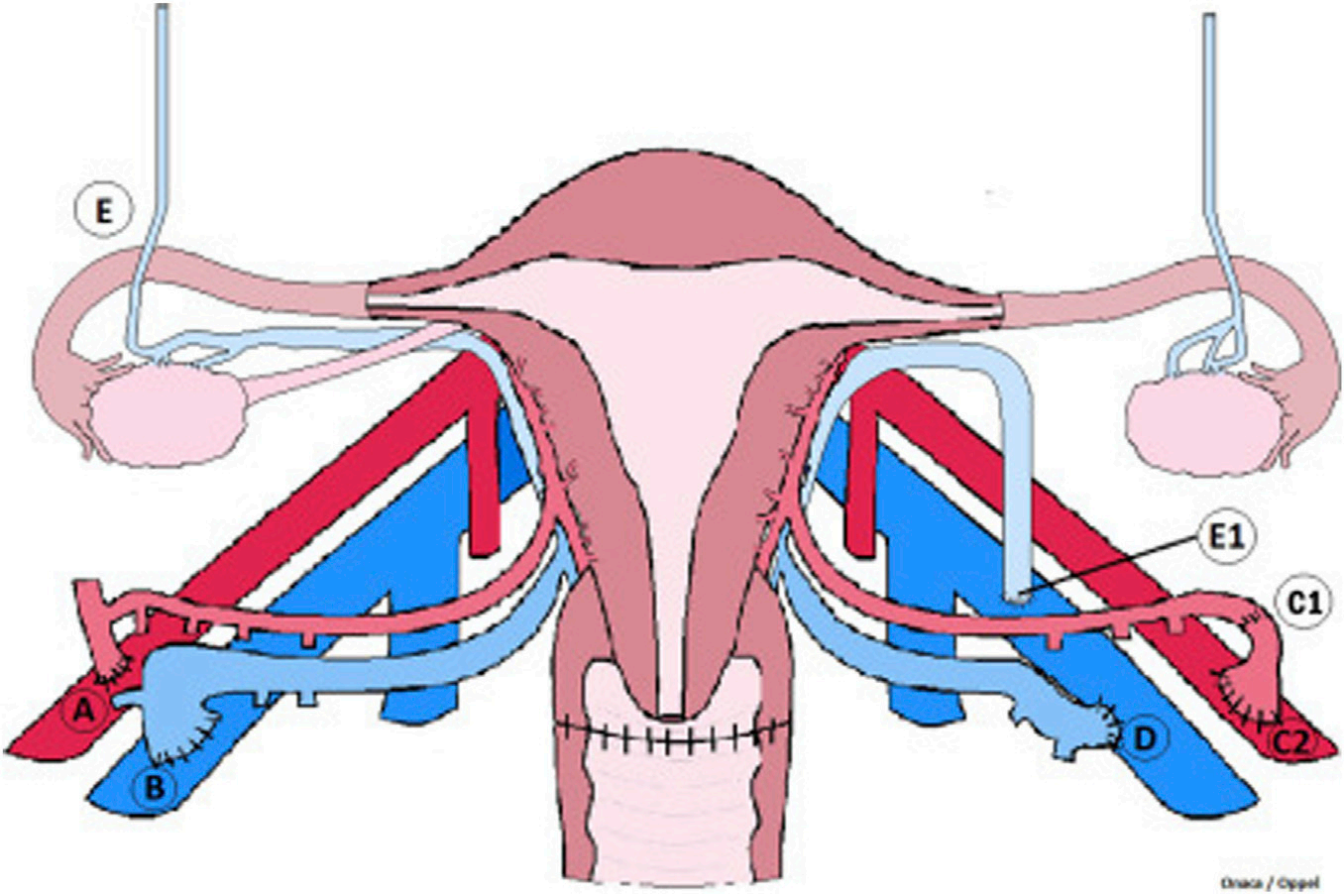
Vascular connections of a uterus transplant procedure. Illustrated are the vascular connections made during graft placement. (A) illustrates anastomosis between internal iliac segment in continuity with uterine artery and external iliac artery. (B) illustrates anastomosis of the uterine vein and external iliac vein with venotomy performed as a simple slit at the superior aspect of iliac vein. (C1) represents a reconstructed uterine artery end-to-end with pudenal artery branch. (C2) shows the internal iliac artery patch end to side with the external iliac artery. (D) demonstrates modified vein anastomosis of the external iliac vein venotomy performed at the medial aspect of the vein as a large oval orifice. (E) shows ovaries fallopian tubes and utero-ovarian vessels. (E1) illustrates anastomosis of utero-ovarian vein and external iliac vein with venotomy performed as a simple slit at the superior aspect of iliac vein. This figure was initially published in the article “Living Donor Uterus Transplantation: A Single Center’s Observations and Lessons Learned From Early Setbacks to Technical Success”.

The second part of the surgery, the back table procedure, follows the donor hysterectomy. The uterine graft is flushed with cold preservation fluid, and vascular reconstruction is performed when necessary [63]. The final and third part of the surgery is the recipient transplantation surgery that starts with the dissection of the external iliac vessels and the top of the vaginal vault. The uterine graft vasculature is thereafter anastomosed bilaterally to the external iliac vessels in the recipient. After graft reperfusion the vaginal rim of the uterine graft is anastomosed to the vaginal vault in the recipient [58, 64]. The connections made in the recipient surgery are illustrated in Figure 5. Since UTx is a temporary transplantation, only meant to stay with the recipient for pregnancy and childbirth, a graft hysterectomy is planned after delivery of 1–3 children. A second and potential third pregnancy is possible if the recipient so wishes, and there are no medical conditions related to complications of immunosuppression or gestational pathologies that would increase the risk for the mother.

### Recipient Care Post Uterus Transplant

The immunosuppression regiment (IR) in UTx recipients is an aspect of care that requires careful consideration. The mainstay of immunosuppressive therapy is not dissimilar from any other solid organ transplant: induction with Thymoglobulin and maintenance with a calcineurin inhibitor, an antimetabolite, and steroids as an addition in some cases. None of these drugs have a profile free of side effects and the goal is to minimize the impact on the wellbeing of the mother and the child. Calcineurin inhibitors are known to be nephrotoxic due to their arteriolar vasoconstrictive effects [65]. In the initial experience with Utx, the antimetabolite of choice in many centers was mycophenolate mofetil (MMF) [32, 33, 36, 37, 66]. MMF was used immediately post-transplant but had to be stopped at least 3 months prior to embryo transfer due to its fetotoxic profile with increased risk of spontaneous abortion and congenital malformations [67–70]. The Dallas team started to completely eliminate MMF and substitute it with Azathioprine, another antimetabolite with a more benign profile, that is started immediately post-surgery [70]. This approach is now utilized by most teams worldwide. The medications used today are safe at therapeutic doses during pregnancy and solid organ transplant recipients have comparable maternal-fetal outcomes to nontransplant patients [71–73].

In UTX recipients, monitoring renal function post-operatively is imperative. Among UTx recipients, 30 percent developed pre-eclampsia which is a risk factor for subsequent kidney injury [74, 75]. Post-transplant, recipients typically see reductions in their glomerular filtration rate (GFR) [74, 76]. However, those who developed pre-eclampsia have sustained reductions in GFR while those that did not develop pre-eclampsia have a return to baseline GFR following withdrawal of immunosuppression [74]. The combination of renal comorbidities that can be congenitally present in MRKH recipients and the need for immunosuppression places UTx recipients at risk for renal dysfunction [77]. Nonetheless, the renal outcomes of UTx recipients should continue to be investigated to ensure safe outcomes, and to provide adequate information during the informed consent process.

**FIGURE 6:**
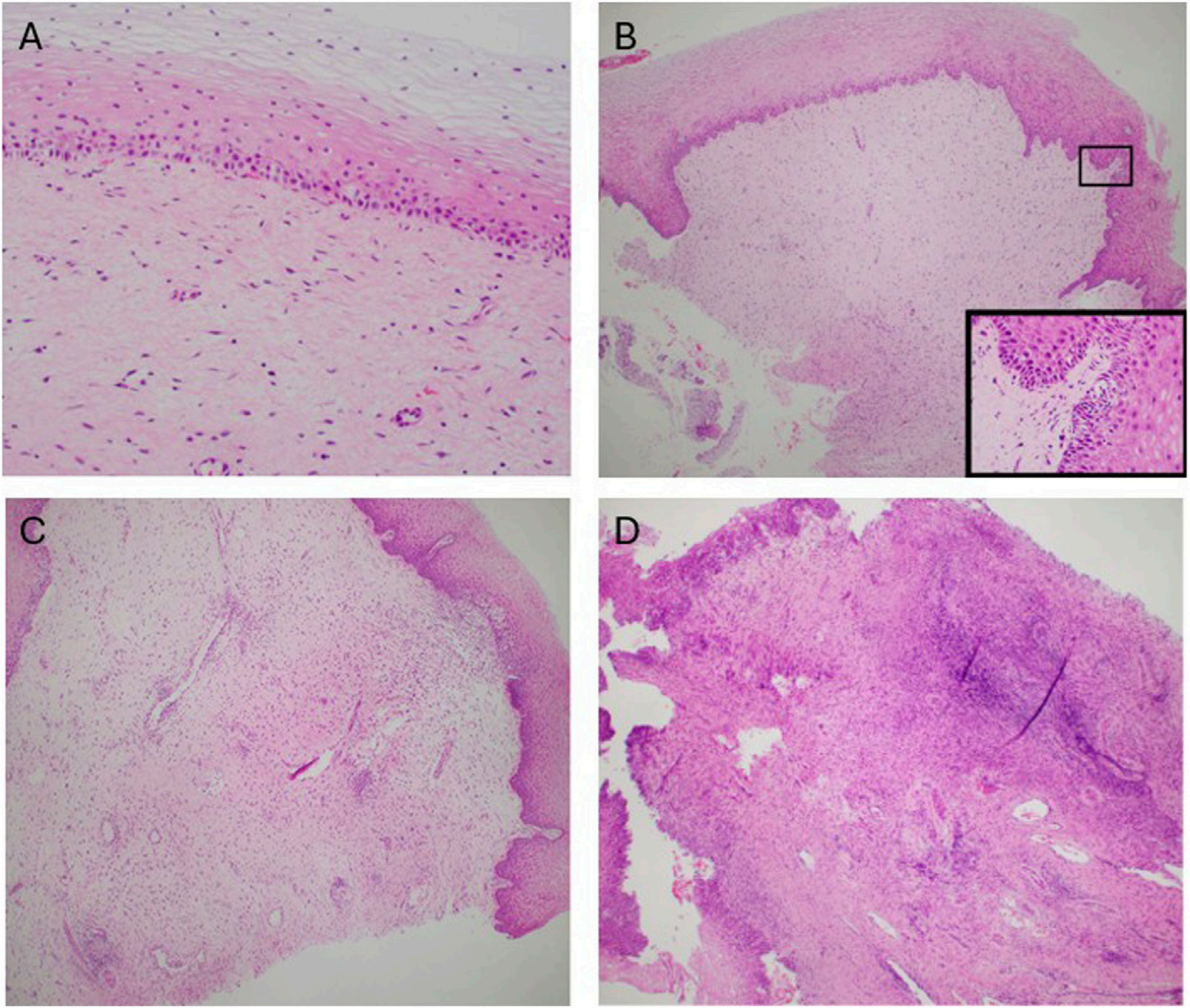
Stages of graft rejection **(A)** No rejection **(B)** Mild rejection **(C)** Moderate rejection **(D)** Severe rejection. This figure was initially published in the article “Clinicopathological Analysis of Uterine Allografts Including Proposed Scoring of Ischemia Reperfusion Injury and T-cell–mediated Rejection—Dallas UtErus Transplant Study: A Pilot Study”.

Episodes of graft rejection in UTx are common and have no clinical manifestations and are diagnosed via cervical biopsy. In addition, there is no serum marker that can assist in the detection or diagnosis of acute cellular rejection. For this reason, frequent monitoring with cervical biopsies are performed [78, 79]. It is only when the acute cellular rejection is not detected and treated that there is progression to clinical signs and symptoms: discoloration of the uterus, increased uterine volume, watery discharge, abdominal pain, and changes in the normal urogenital flora to the presence of beta-hemolytic *streptococcus* Group B [80]. The stages of graft failure are shown in Figure 6. Further investigation into moderate to severe episodes of rejection have identified 13 genes with overlapping expression amongst moderate to severe cases with 5 genes (AGHDIB, BASP1, FCGR3A/B, KLF4, PTPN6) being associated with rejection in other types of organ transplant [81]. Additional investigation in graft rejection has focused on determining non-histological biomarkers that can be used to determine rejection with Keratin 1 granzyme B, IL1β emerging as a potential indicator [82]. However, further investigation amongst larger patient populations is still needed to validate Keratin 1 granzyme B, IL1β′s effectiveness in determining rejection amongst UTx recipients. So far, there are currently no reported cases of graft loss due to treatment resistant acute rejection [83]. Episodes of graft rejection have been shown to be responsive to treatment with corticosteroids with live birth still being possible even after severe episodes of rejection [84, 85].

### *In vitro* Fertilization in Uterus Transplantation

*In vitro* fertilization (IVF) is a necessity for fertilization following UTx since the Fallopian tubes are not included in the uterine graft. Embryos are generated prior to transplant [86]. Amongst centers, there is some variation in the required number of embryos generated with some centers requiring at least 2 and others requiring 6 with IVF treatment and cycle management being left to the discretion of the reproductive endocrinologist [87]. In the event of embryo exhaustion, additional oocyte retrievals can be performed post-transplant, although this will ultimately prolong the time the recipient is on immunosuppressive medication [87]. Currently, reported rates of embryo exhaustion are 20 percent amongst US centers [87]. However, this patient cohort remains too small to generalize across UTx recipients and requires further investigation as the number of UTx recipients grows to determine the standard rate of embryo exhaustion.

In the early days of UTx, embryo transfer was delayed to 1 year post-transplant [84]. The year long wait was based on recommendations for other types of organ transplant by the American Society of Transplantation in 2005 [88]. These recommendations were concerned with many of the same aspects that apply to UTx such as risk of acute rejection, risk of infection that could endanger the fetus, the fetotoxic profile of immunosuppressive medications, and adequate graft function. However, the recommendations made by the American Society of Transplantation were primarily concerned with long-term graft function. UTx is a temporary transplant where a main concern is minimizing a healthy person’s long-term exposure to immunosuppressive medications that could potentially damage their renal function [65]. As a result, transplant centers have elected to shorten their timeframe from UTx to embryo transfer to 3–6 months [86, 89]. The shorter time frame is a patient-centered approach that accounts for graft viability and risk or infection while minimizing the exposure to immunosuppressive medications. The outcome data suggests that it is feasible, safe, and associated with a high implantation rate, to transfer an embryo as early as 3 months after the transplant [89].

### Outcomes of the Children Born After Uterus Transplantation

The long-term outcomes of children born after UTx is limited due to the novelty of the procedure. The longest follow up in the world is 11 years and in the US 8 years [90]. All deliveries have so far been performed via cesarean section due to concerns for vaginal anastomosis dehiscence and the potential for damaging the neovagina and surrounding structures during vaginal labor [29]. Initial experiences in the US have reported a median gestational age at delivery is 36 weeks [29, 91]. No congenital malformations have been recorded [29, 92]. In addition, the median birth weight amongst live births in the US has been reported to be 2,860 g suggesting that low birth weight in UTx may not be as prevalent compared to other forms of organ transplant [93–95]. Long term follow-up of the children has indicated normal neurological and functional development [96]. Overall, the initial long-term outcomes of the children born because of UTx have been favorable. Nonetheless, this remains an ongoing area of research in UTx and additional longitudinal studies are still needed to verify the results seen so far.

## Future Directions of Uterus Transplantation

Currently, the cost of UTx remains a potential barrier to access. Estimates have placed the cost of a single live birth in the US to be $116,137.20, and the total cost per live birth from the Swedish clinical trial being €107,120 [97, 98]. Future efforts to mitigate the costs associated with UTx through insurance coverage can help alleviate this barrier. However, this remains a more complex challenge in healthcare systems like the United States. Nonetheless, future studies on the costs associated with UTx are needed to inform potential recipients and donors fully, and so that conversations regarding potential coverage can occur.

As the field of UTx continues to expand, various aspects still need to be addressed. One such aspect is that the general population’s knowledge of UTx remains relatively low. In a cross-sectional survey, only 33 percent of respondents who were aware of overall organ transplant indicated they had heard of UTx [99]. These results represent how those who may benefit from UTx may not be aware of the procedure, indicating a potential visibility issue, making it difficult to assess overall demand. While provider support has been favorable, determining provider knowledge and awareness in countries and regions without UTx may help increase UTx’s visibility to eligible patient groups as UTx continues to expand in the clinical setting [100]. The further expansion of UTx in a clinical setting also warrants reassessment of patient groups who may not have AUFI, but experience significant challenges in family planning, such as patients with endometriosis. Another similar example is UTx in transgender women. While this topic has been heavily discussed as an additional patient population, there have not been any documented attempted transplants in this patient population [101–103]. Nonetheless, in a study consisting of 186 transgender women, 94 percent agreed or strongly agreed that gestation and childbirth would enhance their self-perception of their femininity [103]. Additionally, nearly all felt that UTx would lead to a greater sense of happiness in male to female transgender women [103]. The results of this study suggest that UTx has significant interest in this currently underserved population. As the field of UTx progresses forward, the inclusion of transgender women has significant potential to expand the pool of potential recipients. Further discussion regarding expanding UTx to this population should focus on the identification of the technical aspects that go into a successful transplant, and identification of barriers to access unique to male to female transgender women.

With the near-horizon expansion of the potential recipient pool, the supply of grafts may need to adjust accordingly. One potential possibility noted is the reuse of uterine grafts or “domino transplants” similar to what has been seen in heart, liver, and kidney transplant [104]. While this is a potential possibility, it will likely remain theoretical. To our knowledge, there have been no attempts, and an attempt would require a significant amount of coincidence and be a significant logistical undertaking, making it an unlikely option for significant meaningful expansion. Instead, a more fruitful option comes in the form of biologically engineered grafts. The current research needed to make this a potential reality is already underway in various different animal models [105, 106]. The significance of biologically engineered grafts is that it nullifies both the challenges associated with deceased donation and the risk of potential complications in living donors. However, before integration in human transplant, a significant amount of further testing is needed to ensure its safety and validity.

## Conclusion

While there remain aspects of UTx that need further assessment and discussion, UTx is an established treatment for AUFI. Coordination and collaboration amongst providers is vital to further expansion of UTx in the clinical setting. As more transplants are performed and additional live births occur, ancillary studies are necessary to build upon the existing knowledge of the field and ensure favorable outcomes.
